# Association between lactate to hematocrit ratio and 30-day all-cause mortality in patients with sepsis: a retrospective analysis of the Medical Information Mart for Intensive Care IV database

**DOI:** 10.3389/fmed.2024.1422883

**Published:** 2024-08-13

**Authors:** Wentao Duan, Feng Yang, Hua Ling, Qiong Li, Xingui Dai

**Affiliations:** Department of Critical Care Medicine, Affiliated Chenzhou Hospital (The First People’s Hospital of Chenzhou), University of South China, Chenzhou, China

**Keywords:** lactate to hematocrit ratio, sepsis, all-cause mortality, MIMIC-IV database, prognosis, retrospective analysis

## Abstract

**Background:**

The lactate to hematocrit ratio (LHR) has not been assessed for predicting all-cause death in sepsis patients. This study aims to evaluate the relationship between LHR and 30-day all-cause mortality in sepsis patients.

**Methods:**

This retrospective study used the data from Medical information mart for intensive care IV (MIMIC-IV, version 2.0). Our study focused on adult sepsis patients who were initially hospitalized in the Intensive care unit (ICU). The prognostic significance of admission LHR for 30-day all-cause mortality was evaluated using a multivariate Cox regression model, ROC curve analysis, Kaplan–Meier curves, and subgroup analyses.

**Results:**

A total of 3,829 sepsis patients participated in this study. Among the cohort, 8.5% of individuals died within of 30 days (*p* < 0.001). The area under the curve (AUC) for LHR was 74.50% (95% CI: 71.6–77.50%), higher than arterial blood lactate (AUC = 71.30%), hematocrit (AUC = 64.80%), and shows no significant disadvantage compared to qSOFA, SOFA, and SAPS II. We further evaluated combining LHR with qSOFA score to predict mortality in sepsis patients, which shows more clinical significance. ROC curve analysis showed that 6.538 was the optimal cutoff value for survival and non-survival groups. With LHR ≥6.538 vs. LHR <6.538 (*p* < 0.001). Subgroup analysis showed significant interactions between LHR, age, sex, and simultaneous acute respiratory failure (*p* = 0.001–0.005).

**Conclusion:**

LHR is an independent predictor of all-cause mortality in sepsis patients after admission, with superior predictive ability compared to blood lactate or hematocrit alone.

## 1 Introduction

Sepsis is considered a life-threatening organ dysfunction caused by a dysregulated response to infection (1). It results in 11 million deaths globally every year, accounting for one-fifth of all death causes (2). In China alone, 1,025,997 deaths related to sepsis were reported in 2015 (3), imposing a substantial burden on public health and economic development. Therefore, effective early prediction of prognosis in sepsis patients holds excellent value.

Previous studies have proposed various parameters for predicting early prognosis in sepsis patients. Blood lactate, an easily accessible laboratory parameter, has been widely used in clinical practice to predict risk factors for mortality in sepsis patients (4, 5). Generally, patients with blood lactate >2 mmol/L within 24 h of admission have an increased mortality rate during ICU stay (6). High lactate can be treated by treating systemic tissue hypoxia, thereby reducing mortality (7). Hence, monitoring lactate levels is crucial in sepsis treatment. Lactate levels not only promptly assess the severity of the condition but also guide treatment adjustments, improving treatment outcomes and reducing mortality rates (8). Recent studies have shown that lactate plays a significant role in predicting patient survival and resuscitation markers (9).

In sepsis patients, intense inflammatory reactions caused by bacterial infections can lead to changes in blood components, such as white and red blood cells (10). Studies have shown that changes in hematocrit (HCT) in sepsis patients are closely related to disease severity and prognosis (11). These changes may be due to red blood cell damage and dissolution caused by inflammatory reactions and blood dilution caused by fluid resuscitation. Therefore, monitoring hematocrit changes is crucial during sepsis treatment, allowing timely assessment of treatment efficacy and adjustment of treatment plans to avoid fluid overload or inadequate volume. Changes in hematocrit can also serve as an important prognostic indicator for sepsis patients. Low HCT levels are an independent risk factor for increased 30-day mortality in sepsis patients, serving as an important predictive indicator for clinical outcomes in sepsis (11).

Recently, many studies have proposed new composite indices to predict the inpatient mortality rate of sepsis patients, such as the lactate/albumin ratio (12), glucose/lymphocyte ratio (13), neutrophil/lymphocyte ratio (14), and platelet/lymphocyte ratio (15).

Given the above information, using lactate and hematocrit levels as combined parameters to predict mortality in sepsis patients may be more meaningful. Previous studies have preliminarily demonstrated the role of lactate to hematocrit ratio (LHR) in predicting mortality rates in patients with severe thoracoabdominal trauma (16). However, no study has evaluated the prognostic value of blood LHR in sepsis patients. Therefore, this study aims to investigate whether the LHR can be a reliable and accurate indicator for predicting 30-day all-cause mortality in sepsis patients upon admission.

## 2 Methods

### 2.1 Database introduction

The data for this study were obtained from the Medical Information Mart for Intensive Care IV (v2.0) database, an extensive publicly accessible database developed and managed by the MIT Laboratory for Computational Physiology^1^ (17). This database covers all patients admitted to Beth Israel Deaconess Medical Center between 2008 and 2019, including hospitalization duration, laboratory tests, medication treatments, and nursing information. All personal information has been de-identified to protect patient confidentiality, with random codes replacing patient identifiers, thus eliminating the need for patient-informed consent and ethical approval. The MIMIC-IV (v2.0) database is available for download from the PhysioNet online forum.^2^ To access this database, the first author of this study, WD, completed the Collaborative Institutional Training Initiative course and passed the “Conflicts of Interest” and “Only Data or Specimen Research” exams (ID: 51905188). Consequently, the research team obtained the qualifications to use and extract data from the database.

### 2.2 Patient selection criteria

**Figure 1:**
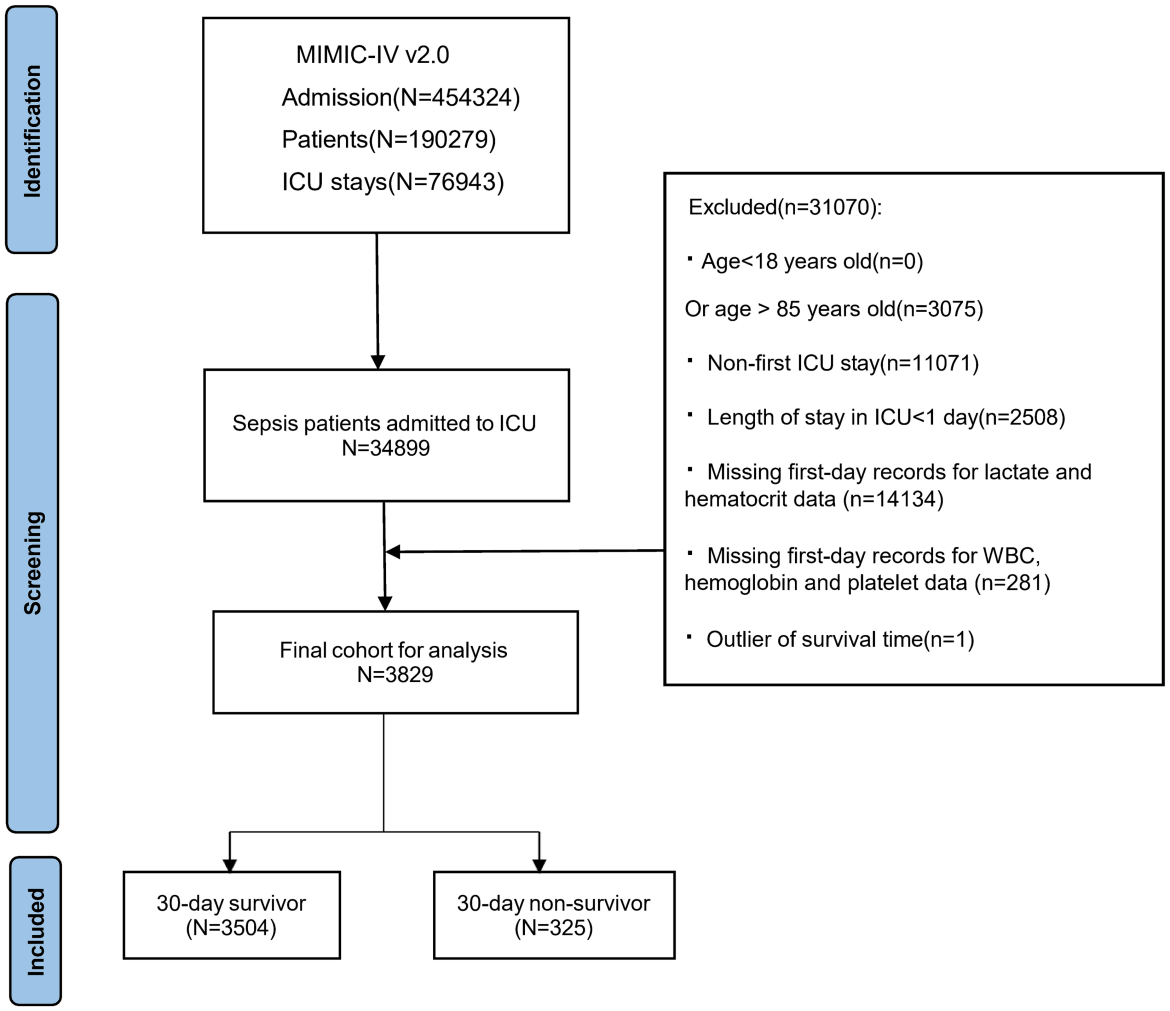
Patient selection flowchart, MIMIC, Medical information mart for intensive care; ICU, Intensive care unit.

The MIMIC-IV database contains records of 454,324 hospitalized patients, of whom 76,943 were admitted to the ICU. Sepsis was defined as a ≥2-point increase in Sequential Organ Failure Assessment (SOFA) score, accompanied by a confirmed or suspected infection (details in Supplementary material). Following the sepsis diagnostic criteria of the SPESIS-3 international standard, hospitalization information of sepsis patients, including 34,899 ICU admission patients, was extracted. Following further screening, patients meeting the following criteria were excluded: (1) patients aged below 18 or above 85 at initial admission; (2) sepsis patients with multiple admissions, retaining data from the first admission only; (3) patients with ICU stay durations of less than 24 h; (4) patients with no recorded data for blood lactate and hematocrit within 24 h of admission. The final cohort included 3,829 patients, as illustrated in Figure 1, depicting the inclusion flowchart.

### 2.3 Data extraction

The included variables comprise age, gender; nursing information (systolic blood pressure, diastolic blood pressure, mean arterial pressure, heart rate, respiratory rate, CVP, intake and output volume), laboratory results (glucose, white blood cell count, hemoglobin, hematocrit, platelet count, sodium, potassium, bicarbonate, lactate, blood urea nitrogen, creatinine), severity at admission [assessed by simplified Acute Physiology Score (SAPS) II], Charlson comorbidity index (CCI), SOFA score, qSOFA score, interventions [mechanical ventilation, continuous renal replacement therapy (CRRT), use of vasoactive medications (dopamine, dobutamine, norepinephrine, epinephrine and vasopressin) at admission], comorbidities (acute heart failure, acute respiratory failure, acute kidney failure, septic shock) and infection site (categorized as unknow, respiratory system infection, abdominal infection, skin or surface infection, bone or joint infection, bloodstream infection, nervous system infection, urinary system infection, intravenous catheter related infection, unclassifiable infection and mixed infection). Additionally, we determined the sites of infection primarily based on specimens collected for pathogen analysis. Due to the inability to classify some specimens into specific systems, we categorized them as unclassifiable infections (Supplementary Table S1 for details). We extracted nursing information (including SBP, DBP, MAP, RR, HR, intake and output volume), laboratory results, and scoring system values (SOFA and qSOFA) recorded within 24 h of admission. Due to the high rate of missing values for central venous pressure (CVP), we used the first recorded CVP measurement to minimize missing data. LHR is calculated using lactate (mmol/L) divide by hematocrit (%). The data was extracted using Navicat Premium software (version 15), employing SQL (Structured Query Language) for the process. All codes used for statistical analysis of patient characteristics, laboratory indicators, complications, and severity scores were obtained from the GitHub repository (MIT-LCP/mimic-iv). For the latest MIMIC-IV code, please refer to the GitHub page: https://github.com/MIT-LCP/mimic-code.

### 2.4 Statistical analysis

The baseline characteristics of the study population were grouped according to different outcome events. Continuous variables are presented as mean ± standard deviation or median (interquartile range), while categorical variables are presented as numbers (percentages). A one-way ANOVA analysis of variance or rank-sum test was used to compare continuous variables in the baseline characteristics analysis of study subjects; the chi-square test and Fisher’s test were used to compare categorical variables.

Univariate Cox regression analysis was used in this study to determine potential risk factors, and variables with *p*-values less than 0.05 were included in the multivariate Cox regression analysis to identify independent risk factors for inpatient mortality. Subsequently, receiver operating characteristic (ROC) analysis was used to evaluate the predictive ability of lactate, hematocrit, LHR, SOFA, qSOFA and SAPS II at admission for mortality rate, as well as the sensitivity and specificity of each indicator, and the area under the curve (AUC) was calculated. Using the Youden index, the optimal cutoff value of LHR was determined; LHR was divided into high and low groups. The Kaplan–Meier (KM) Curve method was then used to plot unadjusted survival curves, and the log-rank test was used to compare the two curves. Subgroup analysis investigated whether LHR impacted different subgroups (including age, acute heart failure, acute respiratory failure, septic shock, obesity, etc.). All analyses were performed using the Free Statistics Software v1.7.1,^3^ the statistical package R v4.2.2 (http://www.R-project.org, the R Foundation) and MedCalc® Statistical Software version 20 (MedCalc Software Ltd., Ostend, Belgium; https://www.medcalc.org; 2021). A two-tailed test with *p* < 0.05 was considered statistically significant.

### 2.5 Management of missing data and outliers

For missing value data, the missing rates of variables such as systolic blood pressure, diastolic blood pressure, mean arterial pressure, heart rate, respiratory rate, platelet count, and creatinine were shallow (0.8, 0.8, 0.15, 0.15, 0.15, 0.23, 0.05%, respectively). However, there were relatively high missing rates for CVP (40.1%), CRRT (94%), vasoactive medications [such as dobutamine (33%), dopamine (33%), epinephrine (33%), norepinephrine (33%), and vasopressin], so they were not included in further analysis and only presented in the baseline characteristics table. As for outliers, we excluded data from one patient with a negative survival time. In the extracted intake and output data, 5.8% of patients had a first-day intake exceeding 10,000 mL, while 62.9% had an intake of 0 mL. These discrepancies made it difficult to assess the patients’ actual fluid therapy accurately. Additionally, 0.71% of patients had first day output exceeding 10,000 mL. Based on our data extraction, approximately 68.9% of patients were diagnosed with anuria and 5.06% with oliguria, assuming output was roughly equivalent to urine output. Therefore, we should have excluded intake and output data in further analysis.

## 3 Result

### 3.1 Baseline demographic and clinical characteristics

Table 1 presents the baseline characteristics of the survival and non-survival groups at 30 day post-admission. A total of 3,829 patients met the inclusion criteria, including 1,371 females (35.8%) and 2,458 males (64.2%). The median age of the patients was 66.5 years (interquartile range, 56.8–75.0 years). The 30-day mortality rate after admission was 8.5%. Compared to the 30-day survival group, we observed that patients in the sepsis non-survival group had lower systolic and mean arterial pressures but higher heart rate, respiratory rate, SAPS II, qSOFA and SOFA scores. CRRT, dobutamine, dopamine, epinephrine, norepinephrine, vasopressin, and mechanical ventilation were used in 89.5, 8.7, 10.5, 27.5, 80.8, 54, and 74.8% of the death group patients, respectively. Additionally, acute heart failure, acute respiratory failure, acute kidney injury, and septic shock were present in 13.5, 51.4, 94.2, and 32% of the non-survival group patients, respectively. Laboratory indicators showed that the LHR at admission was higher in the non-survival group than in the survival group [7.4 (4.4, 14.6) vs. 4.0 (3.0, 5.7), *p* < 0.001]. Blood glucose, CVP, white blood cell count, creatinine, urea nitrogen, lactate, and potassium were significantly higher in the death group (*p* < 0.05). In contrast, hemoglobin, hematocrit, platelets, blood sodium, and bicarbonate were significantly lower in the non-survival group (*p* < 0.05). Additionally, we found that the majority of sepsis patients died from bloodstream infections and mixed infections. The two groups had no statistically significant differences in other covariates (*p* > 0.05).

**Table 1:** Baseline characteristics of participants and outcome parameters.

Variables	Total	Survival	Non-survival	*p*-value
	(*n* = 3,829)	(*n* = 3,504)	(*n* = 325)	
Age, year	66.5 (56.8, 75.0)	66.5 (56.9, 74.8)	67.2 (56.2, 76.4)	0.236
Gender, *n* (%)				0.006
F	1,371 (35.8)	1,232 (35.2)	139 (42.8)	
M	2,458 (64.2)	2,272 (64.8)	186 (57.2)	
**Nursing information**				
SBP, mmHg	115.0 ± 13.3	115.4 ± 13.1	111.7 ± 14.7	<0.001
DBP, mmHg	59.5 ± 8.9	59.5 ± 8.8	59.6 ± 10.1	0.938
MAP, mmHg	76.8 ± 8.9	76.9 ± 8.8	75.4 ± 9.5	0.004
HR, bpm	85.7 ± 14.2	85.2 ± 13.8	91.2 ± 16.9	<0.001
RR, bpm	18.3 ± 3.3	18.1 ± 3.1	20.5 ± 4.5	<0.001
CVP, mmHg	11.0 (8.0, 14.0)	11.0 (8.0, 14.0)	13.0 (9.0, 16.0)	0.001
Intake Volume	0.0 (0.0, 2636.0)	0.0 (0.0, 2318.0)	675.1 (0.0, 4876.0)	<0.001
Output volume	0.0 (0.0, 850.0)	0.0 (0.0, 776.5)	100 (0.0, 1367.0)	<0.001
**Laboratory results**				
Glucose, g/dL	126.0 (102.0, 163.0)	126.0 (102.0, 162.0)	136.0 (107.0, 185.0)	<0.001
WBC, *10^9^/L	8.6 (6.5, 12.2)	8.5 (6.5, 11.8)	11.1 (7.4, 15.8)	<0.001
Hemoglobin, g/L	11.9 ± 2.2	12.0 ± 2.2	10.7 ± 2.4	<0.001
Hematocrit, %	34.5 ± 6.5	34.8 ± 6.4	31.4 ± 7.2	<0.001
Platelet, *10^9^/L	203.0 (153.5, 256.0)	203.0 (156.0, 255.0)	191.0 (126.0, 257.0)	0.006
Sodium, mmol/L	138.7 ± 4.0	138.8 ± 3.8	137.9 ± 6.0	<0.001
Potassium, mmol/L	4.2 ± 0.6	4.2 ± 0.6	4.3 ± 0.9	0.003
Bicarbonate, mmol/L	24.3 ± 4.3	24.5 ± 4.0	21.5 ± 5.7	<0.001
BUN, mg/dL	18.0 (14.0, 25.0)	18.0 (14.0, 24.0)	23.0 (15.0, 37.0)	<0.001
Creatinine, mg/dL	1.0 (0.8, 1.3)	1.0 (0.8, 1.2)	1.1 (0.8, 1.7)	<0.001
Lactate, mmol/L	1.4 (1.1, 2.0)	1.4 (1.0, 1.9)	2.3 (1.4, 4.4)	<0.001
LHR	4.1 (3.0, 6.2)	4.0 (3.0, 5.7)	7.4 (4.4, 14.6)	<0.001
**Score system, points**				
SOFA	6.0 (4.0, 8.0)	5.0 (4.0, 8.0)	13.0 (9.0, 16.0)	<0.001
qSOFA	1.0 (0.0, 1.0)	0.0 (0.0, 1.0)	1.0 (1.0, 2.0)	<0.001
SAPS II	36.0 (29.0, 45.0)	35.0 (28.0, 44.0)	47.0 (38.0, 57.0)	<0.001
CCI	5.0 (4.0, 7.0)	5.0 (4.0, 7.0)	7.0 (5.0, 9.0)	<0.001
GCS	14.0 (10.0, 15.0)	14.0 (11.0, 15.0)	10.0 (3.0, 15.0)	
**Intervention**				
CRRT, *n* (%)				<0.001
No	86 (31.2)	76 (42)	10 (10.5)	
Yes	190 (68.8)	105 (58)	85 (89.5)	
Ventilator use, *n* (%)				<0.001
No	1,310 (34.2)	1,228 (35)	82 (25.2)	
Yes	2,519 (65.8)	2,276 (65)	243 (74.8)	
Dobutamine use, *n* (%)				<0.001
No	2,495 (97.3)	2,243 (98.1)	252 (91.3)	
Yes	68 (2.7)	44 (1.9)	24 (8.7)	
Dopamine use, *n* (%)				<0.001
No	2,468 (96.3)	2,221 (97.1)	247 (89.5)	
Yes	95 (3.7)	66 (2.9)	29 (10.5)	
Epinephrine use, *n* (%)				0.011
No	2009 (78.4)	1809 (79.1)	200 (72.5)	
Yes	554 (21.6)	478 (20.9)	76 (27.5)	
Norepinephrine use, *n* (%)				<0.001
No	1,629 (63.6)	1,576 (68.9)	53 (19.2)	
Yes	934 (36.4)	711 (31.1)	223 (80.8)	
Vasopressin use, *n* (%)				<0.001
No	2,142 (83.6)	2015 (88.1)	127 (46)	
Yes	421 (16.4)	272 (11.9)	149 (54)	
**Comorbidity**				
Acute heart failure, *n* (%)				0.025
No	3,447 (90.0)	3,166 (90.4)	281 (86.5)	
Yes	382 (10.0)	338 (9.6)	44 (13.5)	
Acute respiratory failure, *n* (%)				<0.001
No	3,231 (84.4)	3,073 (87.7)	158 (48.6)	
Yes	598 (15.6)	431 (12.3)	167 (51.4)	
Acute kidney failure, *n* (%)				<0.001
No	755 (19.7)	736 (21)	19 (5.8)	
Yes	3,074 (80.3)	2,768 (79)	306 (94.2)	
Septic shock, *n* (%)				<0.001
No	3,547 (92.6)	3,326 (94.9)	221 (68)	
Yes	282 (7.4)	178 (5.1)	104 (32)	
**Infection site**				
Infection foci, *n* (%)				<0.001
Unknow	1 (0)	1 (0)	0 (0)	
Respiratory system	51 (1.3)	39 (1.1)	12 (3.7)	
Abdominal infection	17 (0.4)	16 (0.5)	1 (0.3)	
Skin or surface infection	7 (0.2)	7 (0.2)	0 (0)	
Bone or joint infection	2 (0.1)	2 (0.1)	0 (0)	
Bloodstream infections	247 (6.5)	188 (5.4)	59 (18.2)	
Nervous system	3 (0.1)	2 (0.1)	1 (0.3)	
Urinary system	549 (14.3)	543 (15.5)	6 (1.8)	
Intravenous catheter related infections	1 (0.0)	1 (0)	0 (0)	
Unclassifiable	1913 (50)	1821 (52)	92 (28.3)	
Mixed infection	1,038 (27.1)	884 (25.2)	154 (47.4)	

Data are presented as the mean ± standard deviation (SD), median (IQR) for skewed variables, and numbers (proportions) or categorical variables. SBP, systolic blood pressure; DBP, diastolic blood pressure; MAP, mean arterial pressure; HR, heart rate; RR, respiratory rate, bpm, beats per minute; WBC, white blood count; BUN, blood urea nitrogen; LHR, lactate to hematocrit ratio; SOFA, Sequential Organ Failure Assessment; qSOFA, quickly Sequential Organ Failure Assessment; SAPS II, simplified Acute Physiology Score II; CCI, Charlson comorbidity index; GCS, Glasgow Coma Scale; CRRT, continuous renal replacement treatment.

### 3.2 Univariate and multivariate Cox regression analysis

We conducted univariate Cox regression analysis for covariates with significant differences (*p* < 0.05) in Table 1. We found that unadjusted LHR was significantly associated with all-cause mortality within 30 days post-admission [hazard ratios (HR) = 1.62, 95% CI: 1.53–1.70, *p* < 0.001]. Additionally, covariates with *p* < 0.05 (Table 2) and potential risk factors were included in the multivariate Cox regression analysis, constructing three models to study the independent impact of LHR on inpatient mortality (more details in multivariable Cox regression models, Table 3). In all three models, both unadjusted and adjusted HRs demonstrated robustness (*p* < 0.05). The unadjusted model showed that with each unit increase in LHR, the difference in inpatient mortality increased by 62% (HR = 1.62, 95% CI: 1.53–1.70). In the minimally adjusted model (Model 1), with each unit increase in LHR, the difference in inpatient mortality increased by 42% (HR = 1.42, 95% CI: 1.34–1.51). In the fully adjusted model (Model 3) (adjusting for covariates including sex, systolic blood pressure, mean arterial pressure, heart rate, respiratory rate, white blood cell count, hemoglobin, blood urea nitrogen, creatinine, blood sodium, blood potassium, bicarbonate, blood glucose, SOFA, SAPS II, CCI, use of mechanical ventilation, comorbidities such as acute heart failure, acute respiratory failure, acute kidney injury, and septic shock and infection site), with each unit increase in LHR, the difference in inpatient mortality increased by 10% (HR = 1.10, 95% CI: 1.01–1.20).

**Table 2:** Univariate Cox regression analysis of risk factors for death within 30 days in patients.

Variables	Univariable Cox		
	HR	HR (95% CI)	*p* (Wald’s test)
Age	1.004	0.9957–1.0123	0.344
*Gender*			
Male	1 (Ref)		
Female	1.36	1.09–1.69	0.006
SBP	0.98	0.97–0.99	<0.001
DBP	1.0006	0.9882–1.0132	0.926
MAP	0.98	0.97–0.99	0.003
HR	1.03	1.02–1.03	<0.001
RR	1.18	1.15–1.21	<0.001
WBC	1.02	1.01–1.02	<0.001
Hemoglobin	0.78	0.74–0.82	<0.001
Platelet	0.999	0.9977–1.0002	0.102
BUN	1.02	1.02–1.02	<0.001
Creatinine	1.21	1.14–1.29	<0.001
Sodium	0.95	0.92–0.97	<0.001
Potassium	1.27	1.1–1.48	0.002
Bicarbonate	0.86	0.84–0.88	<0.001
Glucose	1.003	1.0018–1.0041	<0.001
Lactate	1.28	1.25–1.31	<0.001
Hematocrit	0.93	0.91–0.94	<0.001
SOFA	1.16	1.13–1.18	<0.001
qSOFA	1.83	1.59–2.10	<0.001
SAPS II	1.05	1.04–1.06	<0.001
CCI	1.17	1.13–1.22	<0.001
GCS	0.91	0.89–0.93	<0.001
*Ventilator use*			
No	1 (Ref)		
Yes	1.59	1.24–2.04	<0.001
*Acute heart failure*			
No	1 (Ref)		
Yes	1.43	1.04–1.96	0.028
*Acute respiratory failure*			
No	1 (Ref)		
Yes	6.47	5.21–8.05	<0.001
*Acute kidney failure*			
No	1 (Ref)		
Yes	4.11	2.58–6.53	<0.001
*Septic shock*			
No	1 (Ref)		
Yes	7.05	5.58–8.9	<0.001
Infection foci			<0.001
Mixed infection	1 (Ref)		
Unknow	NA		
Respiratory system	1.79	0.99–3.22	
Abdominal infection	0.38	0.05–2.73	
Skin or surface infection	0	0-Inf	
Bone or joint infection	0	0-Inf	
Bloodstream infections	1.55	1.15–2.1	
Nervous system	4.34	0.61–31.08	
Urinary system	0.14	0.06–0.32	
Intravenous catheter related infections	NA		
Unclassifiable	0.54	0.42–0.7	
LHR	1.62	1.53–1.7	<0.001

**Table 3:** Multivariate Cox regression analysis of risk factors for death in patients within 30 days.

Variable	Unadjusted		Model 1		Model 2		Model 3	
	HR_95 CI%	*p*-value	HR_95 CI%	*p*-value	HR_95 CI%	*p*-value	HR_95 CI%	*p*-value
LHR	1.62 (1.53–1.7)	<0.001	1.42 (1.34–1.51)	<0.001	1.27 (1.17–1.37)	<0.001	1.10 (1.01–1.20)	<0.001

Model 1 adjusted for gender, SBP, MAP, HR, and RR. Model 2 adjusted for Model 1 (+WBC, hemoglobin, bun, creatinine, sodium, potassium, bicarbonate, glucose). Model 3 adjusted for Model 2 (+SOFA, qSOFA SAPS II, CCI, GCS, ventilator use, acute heart failure, acute lung failure, acute kidney failure, septic shock and infection foci).

### 3.3 ROC curve analysis and survival curve

**Figure 2:**
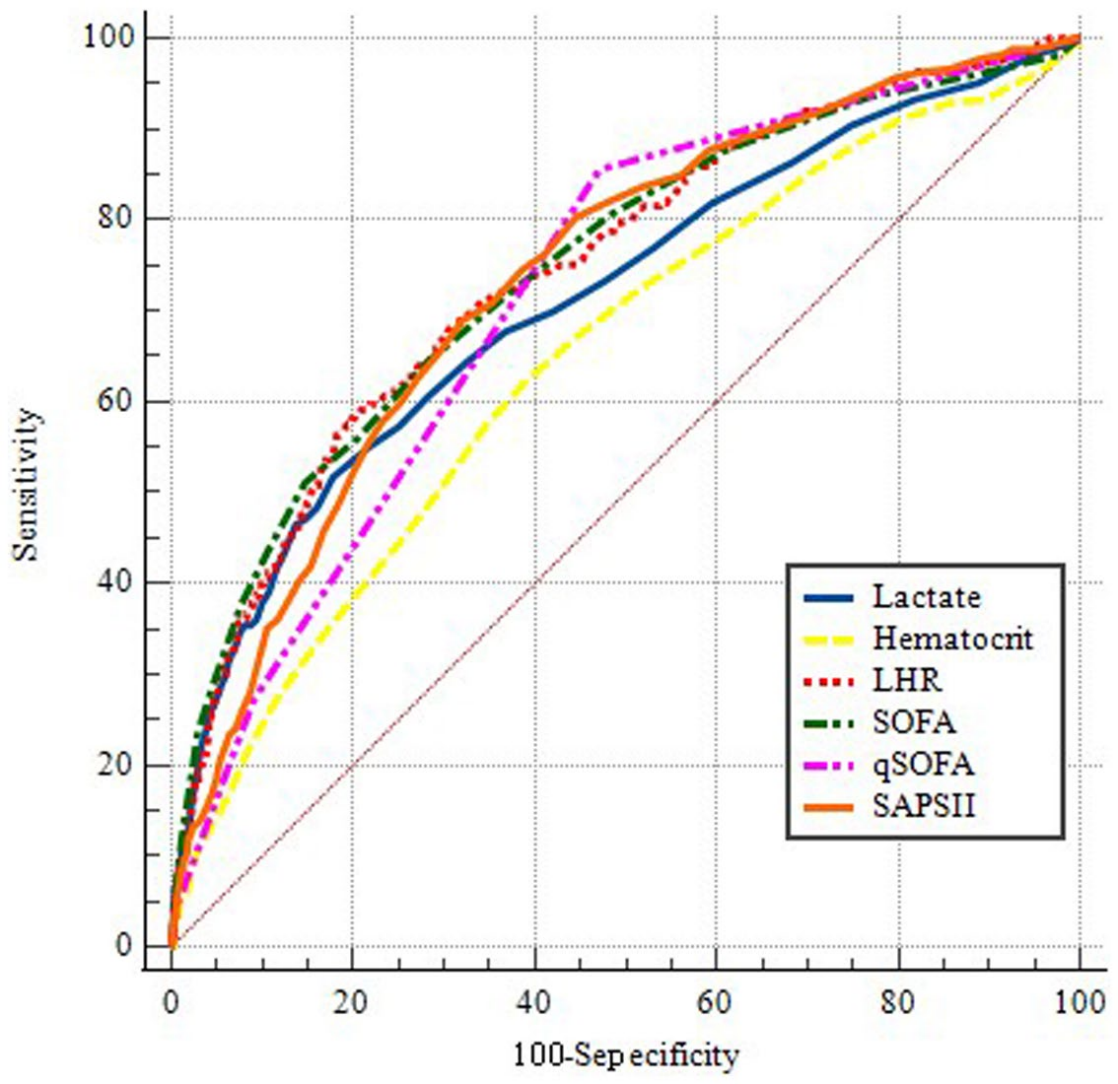
ROC curves for predicting in-hospital mortality. The blue solid line indicates the ROC curve of the lactate. The yellow dotted line indicates the ROC curve for hematocrit. The red dotted line indicates the ROC curve of LHR. The Green dotted line indicates the ROC curve of SOFA. The pink dotted line indicates the ROC curve of qSOFA. The orange solid line indicates the ROC curve of the SAPSII. LHR, lactate to hematocrit ratio. SOFA, Sequential Organ Failure Assessment. qSOFA, quickly Sequential Organ Failure Assessment. SAPS II, simplified Acute Physiology Score II.

**Figure 3:**
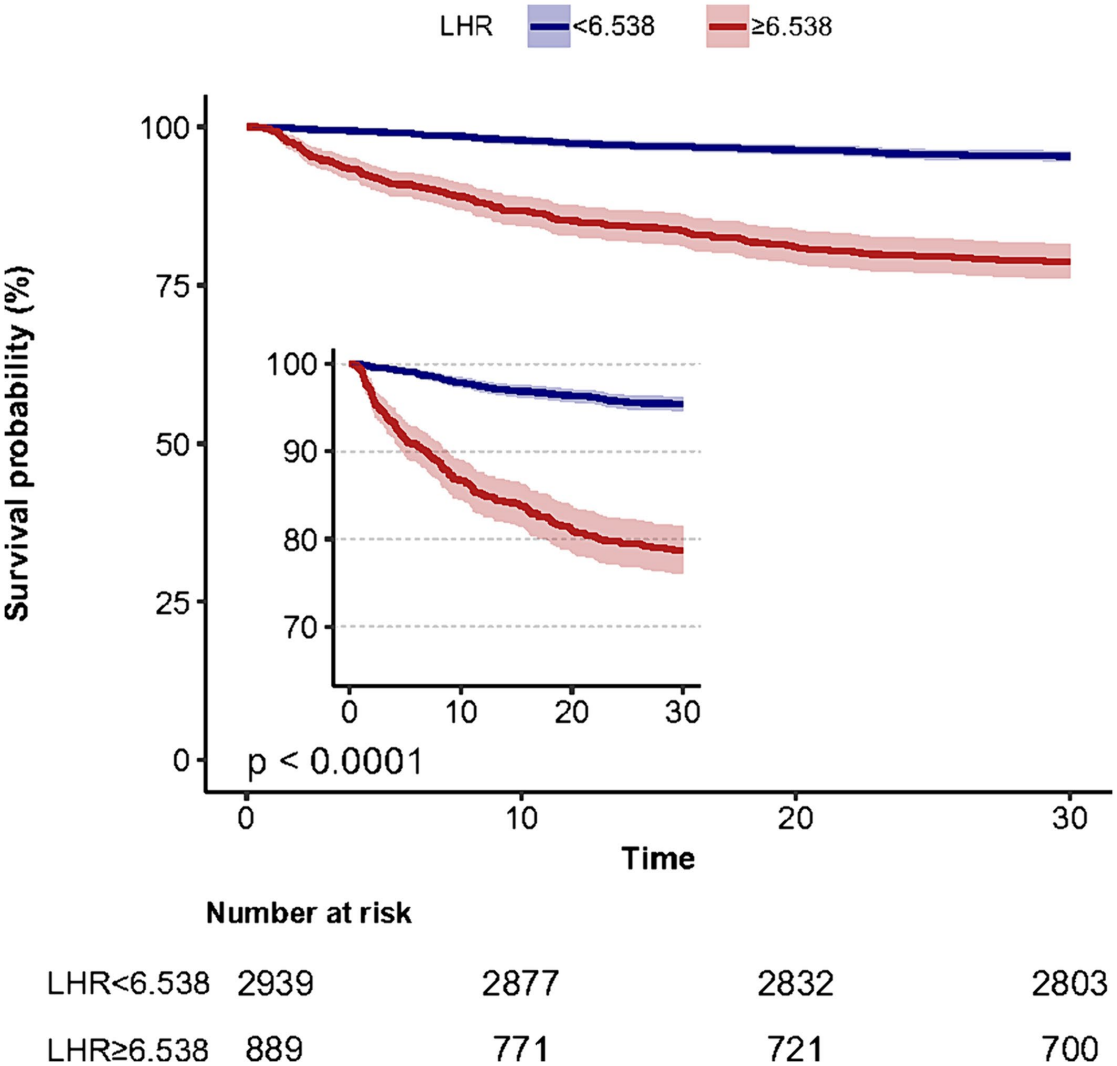
Kaplan-Meier survival analysis curves for all-cause 30-day mortality.

To further assess the predictive value of LHR, we conducted a ROC curve analysis of the 30-day mortality of sepsis patients, including LHR, blood lactate, hematocrit, SOFA, qSOFA and SAPS II socre. The specific ROC curve information is listed in Table 4, and Figure 2 displays the relevant data. The ROC curves (Figure 2) revealed an AUC of 74.5% for LHR (95% CI: 71.6–77.5%), surpassing that of lactate (71.3%), hematocrit (64.8%), both with *p* < 0.001 and LHR showed no significant disadvantage compared to traditional prognostic indicators for sepsis, including qSOFA, SOFA, and SAPS II (*p*-values were 0.1351, 0.9536, and 0.5970, respectively). Therefore, LHR shows a clear predictive advantage. Our study primarily explores the impact of LHR on early mortality in sepsis patients, with ROC curve analysis highlighting the high sensitivity and computational simplicity of the qSOFA score. Thus, combining LHR with qSOFA score to predict mortality in sepsis patients may offer more clinical significance. Further analysis of combined LHR and qSOFA, as well as LHR, SOFA, qSOFA, and APS II scores for prognostic evaluation in sepsis patients (Supplementary Figure S1), showed that LHR combined with qSOFA significantly outperformed using LHR, SOFA, qSOFA, and APS II alone (*p* < 0.001, 0.0028, 0.001, and 0.002, respectively). Moreover, we determined the optimal threshold for LHR to be 6.538, with a sensitivity of 58.15% and specificity of 80.13%. Using this optimal threshold, we categorized sepsis patients into a high LHR group (LHR ≥6.538, *n* = 889) and a low LHR group (LHR <6.538, *n* = 2,940). By plotting KM survival analysis curves (Figure 3), we observed a significantly higher mortality rate in the high LHR group compared to the low LHR group (*p* < 0.001). In addition, we further assessed the impact of LHR on the mid-(Supplementary Figure S2) and long-term (Supplementary Figure S3) prognosis of sepsis patients.

**Table 4:** Information of ROC curves in Figure 2.

Variables	AUC	95% CI	Threshold	Sensitivity	Specificity
LHR + qSOFA	0.793	0.780–0.806	—	—	—
LHR	0.745	0.716–0.775	6.538	0.5815	0.8013
Lactate	0.713	0.681–0.745	2.2	0.5169	0.8224
Hematocrit	0.648	0.616–0.680	33	0.6215	0.6112
SOFA	0.746	0.732–0.760	9	0.5108	0.8533
qSOFA	0.719	0.704–0.733	1	0.8554	0.5288
SAPS II	0.736	0.722–0.750	40	0.6892	0.6807

AUC, area under the curve; CI, confidence interval; ROC, receiver operating characteristic, LHR, lactate to hematocrit ratio, SOFA Sequential Organ Failure Assessment; qSOFA, quickly Sequential Organ Failure Assessment; SAPS II, simplified Acute Physiology Score II.

### 3.4 Subgroup analysis

**Figure 4:**
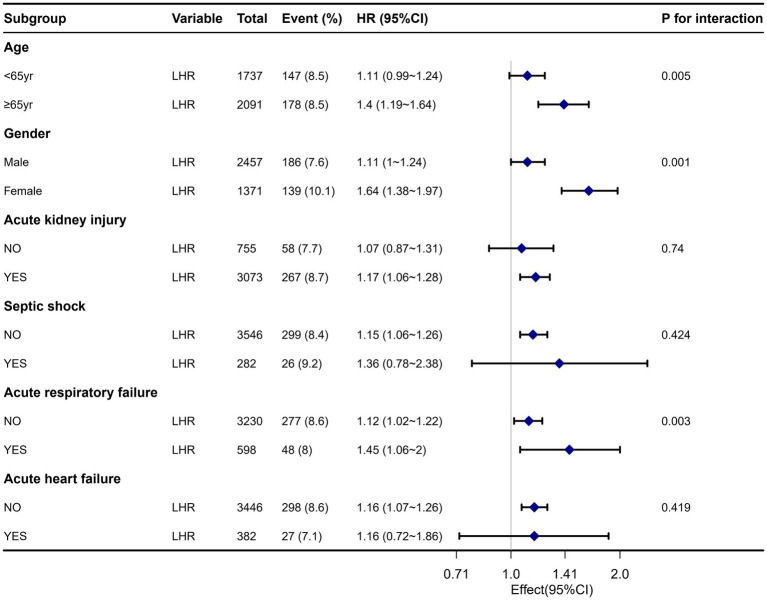
Forest plot for subgroup analysis of the relationship between all-cause mortality and LHR.

Figure 4 displays a robust correlation between LHR and all-cause mortality in sepsis patients across different subgroups at 30 days of hospitalization. We conducted a stratified analysis based on factors such as age, gender, acute kidney injury, septic shock, acute heart failure, and acute respiratory failure. The forest plot (Figure 4) shows significant interactions between LHR and age, gender, and acute respiratory failure (interaction *p*-values were 0.005, 0.001, and 0.003, respectively). Additionally, the subgroup analysis results show a robust correlation between LHR and all-cause mortality across different subgroups.

## 4 Discussion

Using multivariate Cox regression analysis, the study found that LHR is an independent factor for predicting 30-day all-cause mortality in sepsis patients after admission. LHR demonstrated higher predictive accuracy than lactate, hematocrit and combining LHR with qSOFA ROC analysis shows more clinical significance. Furthermore, our KM survival analysis illustrated that patients with LHR ≥6.538 had a significantly higher all-cause mortality within 30 days of admission than those with LHR <6.538; subgroup analysis results supported our findings.

Recently, researchers have extensively explored indicators for predicting prognosis in sepsis patients, including lactate/albumin ratio (12), glucose/lymphocyte ratio (13), neutrophil/lymphocyte ratio (14), and platelet/lymphocyte ratio (15).

Previous studies have preliminarily confirmed the role of lactate and hematocrit ratio in predicting mortality rates in severely injured patients (16). However, using LHR to predict outcomes for sepsis patients remains unreported.

Lactate is an important indicator of tissue oxygenation, blood perfusion, and metabolism, widely used in clinical practice to predict mortality risk in sepsis patients (4, 5). Generally, patients with blood lactate levels exceeding 2 mmol/L within 24 h have an increased mortality rate during ICU admission (6). Early lactate clearance during treatment may indicate alleviation of systemic tissue hypoxia and is associated with reduced mortality rates (7). However, the blood lactate level can be affected by different factors, such as liver disease, malignant tumors, or certain medications (e.g., metformin, catecholamines), However, these factors were not considered in our study. Additionally, some critically ill patients may have lower venous blood lactate levels, reducing the reliability of lactate levels alone in predicting patient outcomes (18).

Sepsis patients often experience microcirculatory disturbances, and fluid resuscitation can prolong patient survival (19). Current guidelines recommend giving at least 30 mL/kg of crystalloid fluids within the first 3 h of sepsis onset for fluid resuscitation (20). However, the intense inflammatory response caused by sepsis can alter vascular permeability (21), leading to progressive tissue and cavity edema, thus increasing the risk of fluid overload with fluid resuscitation. Although fluid resuscitation is adequate for many patients, personalized fluid therapy may be more appropriate, especially for critically ill patients, where the association between fluid administration and mortality rates is more pronounced (22). As an easily obtainable laboratory parameter, hematocrit can reflect blood dilution to a certain extent; however, its association with red blood cells constrains its predictive value as a single factor due to influences from chronic diseases, nutritional support, and inflammation. Therefore, we used the LHR as a composite indicator to more accurately analyze the impact of blood lactate on the prognosis of sepsis patients while considering changes in blood components.

The study by Demir et al. (16) (including 106 patients, AUC for LHR was 0.886, lactate was 0.846) demonstrated that LHR is more predictive of inpatient mortality in severely injured patients than lactate alone. Additionally, the study by Staziaki et al. (23) (including 804 patients) showed that patients with higher blood lactate levels and lower hematocrit levels had higher ICU admission rates and more extended hospital stays in a trauma setting, indirectly reflecting the correlation between changes in blood lactate and hematocrit levels with disease severity. These conclusions are consistent with our study results, where patients in the non-survival group had higher blood lactate levels and lower hematocrit levels than the survival group; based on cutoff value analysis, higher LHR values were associated with increased mortality rates.

Sepsis is defined as a life-threatening organ dysfunction caused by a dysregulated host response to infection. The accompanying maladaptive inflammatory response activates the complement, coagulation, and endothelial systems, leading to microcirculatory disturbances (24), thereby increasing lactate production from anaerobic glycolysis (25). Simultaneously, bacterial and complement activation during sepsis causes intravascular hemolysis (26). Inflammatory cytokines, such as TNF-α, IL-1, IL-2, IL-6, and IL-8, trigger the adhesion of neutrophils and endothelial cells, leading to microthrombus formation (10), thereby reducing the number of red blood cells entering the circulation. Hematocrit levels are affected by the inflammatory response, oxidative stress, and fluid resuscitation-induced blood dilution. Therefore, based on these studies, we further propose a hypothesis regarding the prognostic significance of LHR in sepsis patients.

There are certain constraints in our study. First, the MIMIC-IV database’s overall mortality rate for first-time hospitalized sepsis patients is approximately 17.8% (3,795/21,382). Due to the significant amount of missing data for variables required in this study, such as blood lactate and hematocrit, we employed a method of deleting missing data. This led to the exclusion of some deceased patients, resulting in a lower overall mortality rate in our study cohort. This introduces a selection bias. Additionally. further analysis was not possible due to the substantial amount of missing and anomalous data related to fluid management variables. Although biomarkers such as alanine aminotransferase (ALT) and aspartate aminotransferase (AST) are also proven to be associated with poor prognosis, they were not included in this study due to data inconsistencies and omissions. These factors may reduce the credibility of the study.

In this study, LHR was an independent predictor of all-cause mortality in sepsis patients after admission. Its predictive capability was superior to standalone arterial blood lactate or hematocrit and comparable to SOFA, qSOFA, and SAPS II scores. This indicates that LHR may be a valuable supplement to SOFA in clinical decision-making. Additionally, combining LHR with qSOFA may enhance predictive significance. However, large-scale multicenter prospective studies are still needed to evaluate the effectiveness of LHR comprehensively. Such studies would more accurately validate the role of LHR in sepsis prognosis assessment and provide more reliable evidence for clinical practice.

Lastly, our study data are derived from the MIMIC-IV (v2.0) database, which includes patient information from 2008 to 2019. Due to continuous advancements in medical treatments and optimization of treatment protocols, the extended period may result in inconsistencies in patient treatment plans, potentially biased the study results.

## 5 Conclusion

In this study, LHR was an independent predictor of all-cause mortality in sepsis patients after admission. Its predictive capability was superior to standalone arterial blood lactate or hematocrit and comparable to SOFA, qSOFA, and SAPS II. Additionally, combining LHR with qSOFA may enhance predictive significance. However, large-scale multicenter prospective studies are still needed to evaluate the effectiveness of LHR comprehensively. Such study designs would more accurately validate the role of LHR in sepsis prognosis assessment and provide more reliable evidence for clinical practice.

## Data Availability

The datasets presented in this study can be found in online repositories. The names of the repository/repositories and accession number(s) can be found in the article/Supplementary material.

## Footnotes

^1^ https://physionet.org/content/mimiciv/2.0/

^2^ https://physionet.org/

^3^ http://www.clinicalscientists.cn/freestatistics
