# Supplementary figures and images for: Association between lactate to hematocrit ratio and 30-day all-cause mortality in patients with sepsis: a retrospective analysis of the Medical Information Mart for Intensive Care IV database
Source: Front Med (Lausanne). 2024 Aug 13;11:1422883. doi: 10.3389/fmed.2024.1422883 (PMC11347292; doi:10.3389/fmed.2024.1422883)

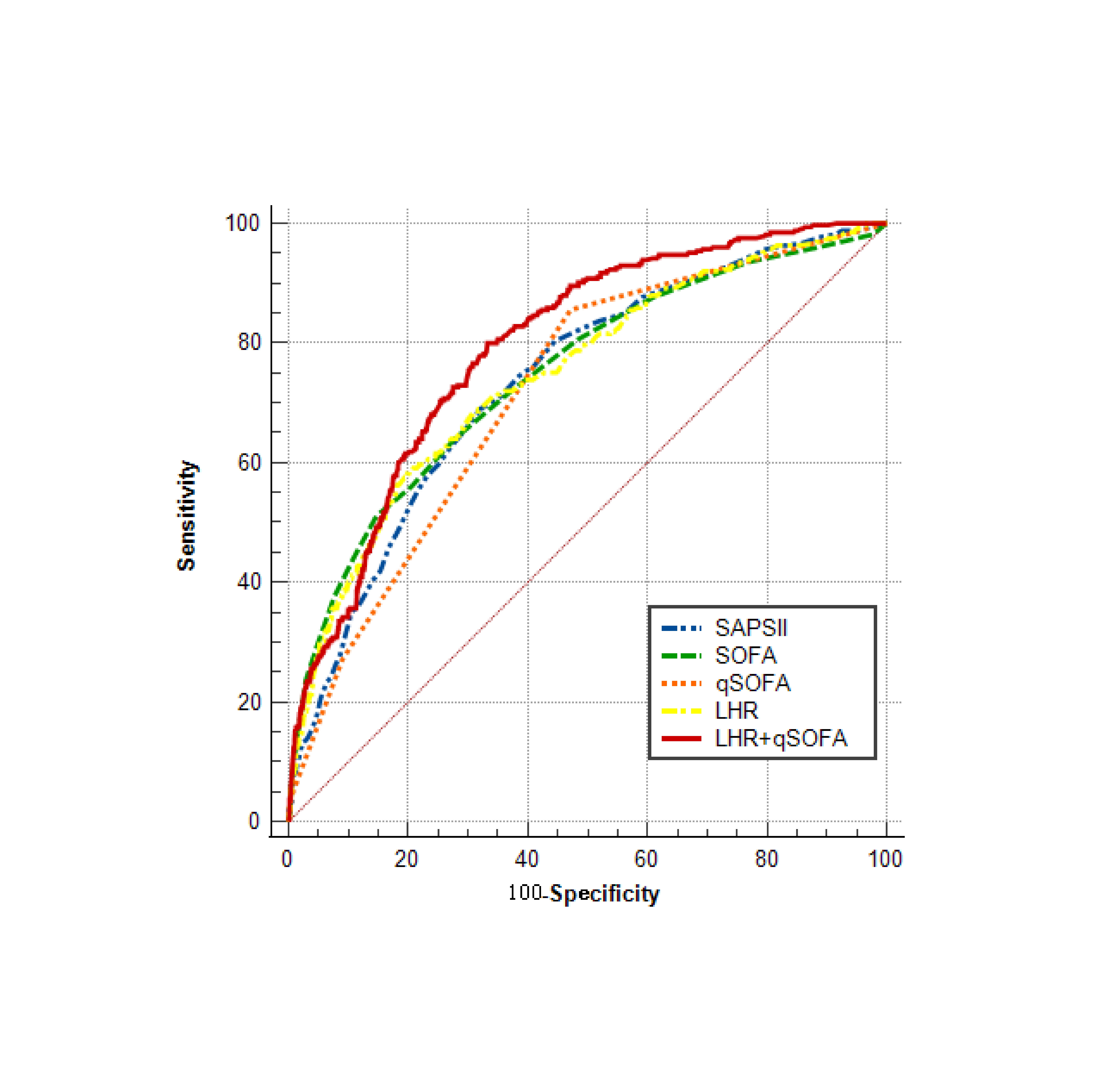

Supplement: Supplementary file 1 [file Image_1.JPEG]

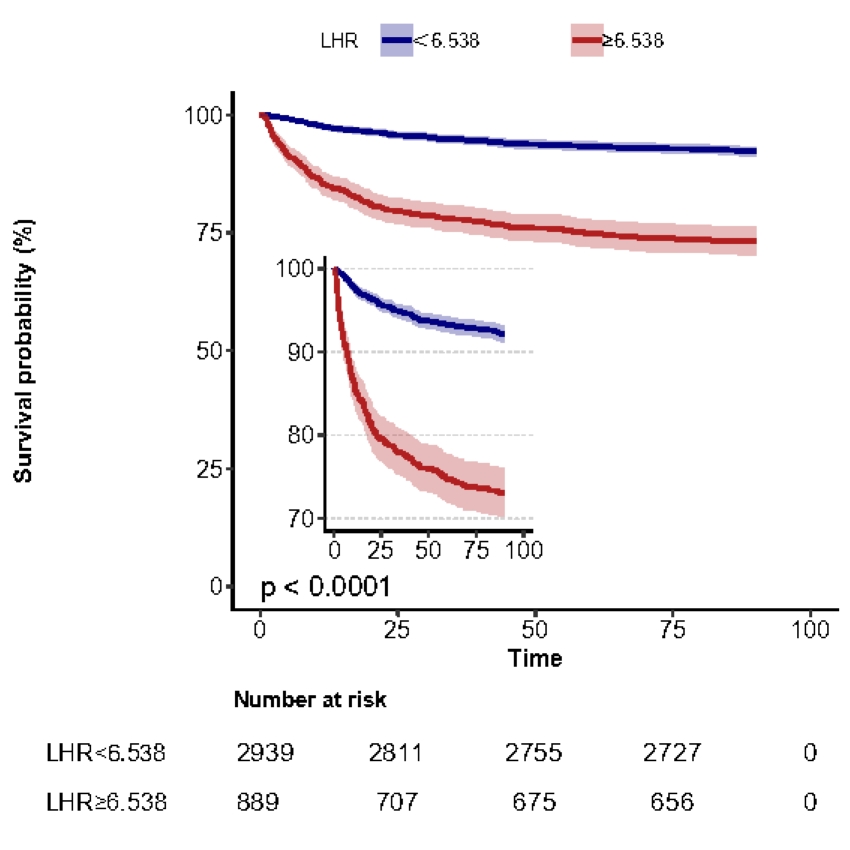

Supplement: Supplementary file 2 [file Image_2.JPEG]

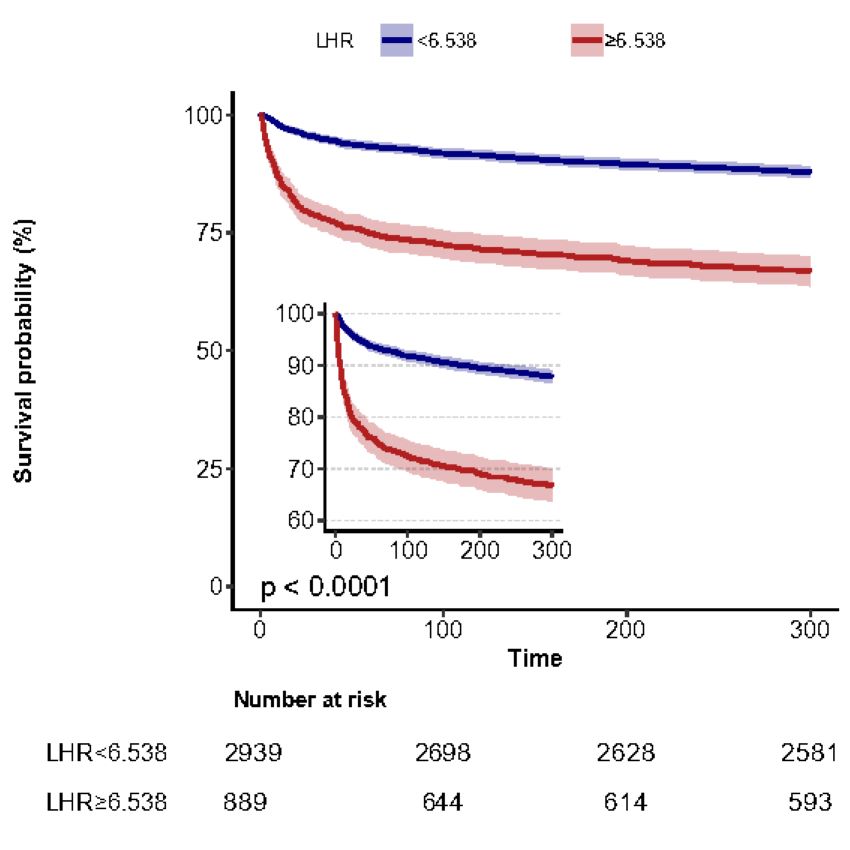

Supplement: Supplementary file 3 [file Image_3.JPEG]
