# Supplementary material for: Association between lactate to hematocrit ratio and 30-day all-cause mortality in patients with sepsis: a retrospective analysis of the Medical Information Mart for Intensive Care IV database
Source: Front Med (Lausanne). 2024 Aug 13;11:1422883. doi: 10.3389/fmed.2024.1422883 (PMC11347292; doi:10.3389/fmed.2024.1422883)
Supplement: Supplementary file 5 [file Table_2.DOCX]

Regarding Sepsis Diagnostic Criteria

i) Definition of Suspected Infection

We define the time of suspected infection as the earlier of either the first administration of antibiotics or the initial culture time (referred to as suspected_infection_time). This timestamp is recorded in the "mimiciv_derived.suspicion_of_infection" table.

ii) Consideration of Infection Time Window

We have defined a time window from 48 hours before to 24 hours after the suspected infection time. This window ensures that we capture changes in SOFA scores before and after the onset of infection, reflecting the progression of sepsis.

iii) Definition of Baseline SOFA Score

We assume a baseline SOFA score of zero before admission. This assumption is made because detailed organ function data before admission is typically unavailable, and most patients do not exhibit severe organ dysfunction upon admission.

iv) Calculation of SOFA Score Changes

Within our defined time window (48 hours before to 24 hours after the suspected infection time), we calculate and record each patient's SOFA score and its components (such as respiratory, coagulation, liver, cardiovascular, central nervous system, and renal). We extract records from the" mimiciv_derived.sofa "table where the SOFA score is equal to or greater than 2, indicating organ dysfunction. The onset time of sepsis can be determined based on either the latest suspected infection time or when the SOFA score first reaches 2 or higher. This approach allows us to identify a cohort of patients diagnosed with sepsis.

The code for extracting the sepsis cohort is available at https://github.com/MIT-LCP/mimic-code/tree/main/mimic-iv/concepts_postgres/sepsis
